# The Long Non-Coding RNA *RP5-1024C24.1* and Its Associated-Gene *MPPED2* Are Down-Regulated in Human Thyroid Neoplasias and Act as Tumour Suppressors

**DOI:** 10.3390/cancers10050146

**Published:** 2018-05-18

**Authors:** Romina Sepe, Simona Pellecchia, Pierre Serra, Daniela D’Angelo, Antonella Federico, Maddalena Raia, Ricardo Cortez Cardoso Penha, Myriam Decaussin-Petrucci, Luigi Del Vecchio, Alfredo Fusco, Pierlorenzo Pallante

**Affiliations:** 1 Institute of Experimental Endocrinology and Oncology (IEOS) “G. Salvatore”, National Research Council (CNR), Via Sergio Pansini 5, 80131 Naples, Italy; romina.sepe@unina.it (R.S.); simona.pellecchia@unina.it (S.P.); daniela.dangelo@unina.it (D.D.); anfederi@unina.it (A.F.); ricardocortezcardoso@gmail.com (R.C.C.P.); 2 Department of Molecular Medicine and Medical Biotechnology (DMMBM), University of Naples “Federico II”, Via Sergio Pansini 5, 80131 Naples, Italy; raia@ceinge.unina.it (M.R.); luigi.delvecchio@unina.it (L.D.V.); 3 Service d’Anatomie et Cytologie Pathologiques, Centre de Biologie Sud, Groupement Hospitalier Lyon Sud, 69495 Pierre Bénite, France; pserra54@gmail.com (P.S.); myriam.decaussin-petrucci@chu-lyon.fr (M.D.-P.); 4 CEINGE-Biotecnologie Avanzate, Via Gaetano Salvatore 486, 80145 Naples, Italy; 5 Instituto Nacional de Cancer, Laboratorio de Carcinogênese Molecular, Rua Andre Cavalcanti 37, Centro, Rio de Janeiro 20231-050, Brazil

**Keywords:** long non-coding RNA, *MPPED2*, thyroid carcinoma, tumour suppressor, carcinogenesis

## Abstract

*Background*: Well-differentiated papillary thyroid carcinoma (PTC) represents the thyroid neoplasia with the highest incidence. Long non-coding RNAs (lncRNAs) have been found deregulated in several human carcinomas, and hence, proposed as potential diagnostic and prognostic markers. Therefore, the aim of our study was to investigate their role in thyroid carcinogenesis. *Methods*: We analysed the lncRNA expression profile of 12 PTC and four normal thyroid tissues through a lncRNA microarray. *Results*: We identified 669 up- and 2470 down-regulated lncRNAs with a fold change >2. Among them, we focused on the down-regulated *RP5-1024C24.1* located in an antisense position with respect to the *MPPED2* gene which codes for a metallophosphoesterase with tumour suppressor activity. Both these genes are down-regulated in benign and malignant thyroid neoplasias. The restoration of *RP5-1024C24.1* expression in thyroid carcinoma cell lines reduced cell proliferation and migration by modulating the PTEN/Akt pathway. Inhibition of thyroid carcinoma cell growth and cell migration ability was also achieved by the *MPPED2* restoration. Interestingly, *RP5-1024C24.1* over-expression is able to increase *MPPED2* expression. *Conclusions*: Taken together, these results demonstrate that *RP5-1024C24.1* and *MPPED2* might be considered as novel tumour suppressor genes whose loss of expression contributes to thyroid carcinogenesis.

## 1. Introduction

Thyroid carcinomas are moderately rare, constituting 1% of all human carcinomas, but represent the highest percentage of all malignancies derived from the endocrine system [1]. Neoplasias resulting from thyroid follicular cells consist of a wide spectrum of lesions with increasing degree of malignancies going from benign follicular thyroid adenomas (FTA), to differentiated carcinomas (papillary thyroid carcinomas, PTC, and follicular thyroid carcinomas, FTC), to completely undifferentiated carcinomas (anaplastic thyroid carcinomas, ATC) that are always fatal [2]. Among thyroid carcinomas, PTC is the most frequent histotype representing about 80% of all diagnosed thyroid carcinomas. Some genetic mutations are already known to be involved in PTC development, such as *RET/PTC* rearrangement [3,4,5], *BRAF* and *RAS* mutations [6,7,8,9]. Moreover, microRNA (miRNA) deregulated expression has been frequently described in human thyroid carcinomas [10]. However, most of the molecular mechanisms underlying thyroid carcinogenesis have not been completely elucidated yet.

In order to better understand the basic mechanisms involved in thyroid carcinogenesis, a great interest has been recently raised by long non-coding RNAs (lncRNAs), single-strand RNA molecules with a length that varies between 200 and 100,000 nucleotides. They are classified in five different groups on the basis of their position in the genome (sense, antisense, bidirectional, intronic, intergenic) [11,12].

Currently, their molecular action mechanisms and their role played in gene expression regulation have not been clarified, but it has been demonstrated that they can modulate gene expression by inducing histonic epigenetic modifications or acting as “sponge” for miRNAs, thus playing a role in both normal biological processes and diseases, including cancer [13,14,15,16,17]. Moreover, recent evidence has shown that lncRNAs are deregulated in several neoplasias depending on the tumour histotype, proposing their detection as an important tool in cancer diagnosis and prognosis [18,19,20].

Therefore, in order to identify deregulated lncRNAs in thyroid neoplasias and unveil their role in thyroid carcinogenesis, we analysed the expression profile of 12 PTC versus four normal thyroid tissues. Several lncRNAs differentially expressed in PTC compared to normal thyroid tissues were identified. Then, we focused on the lncRNA *RP5-1024C24.1* that was also down-regulated in FTA, FTC and ATC. Interestingly, the expression of *MPPED2*, its associated gene, was significantly decreased in the thyroid carcinoma samples analysed, suggesting a role of both these genes in thyroid carcinogenesis. Accordingly, the stable restoration of both *RP5-1024C24.1* and *MPPED2* expression was able to attenuate proliferation and migration rate of thyroid cancer cell lines, suggesting a role of both these genes in thyroid cancer progression.

## 2. Results

### 2.1. Identification of LncRNAs Deregulated in Human PTC

In order to identify the lncRNAs deregulated in PTC, we analysed the lncRNA expression profile of 12 PTC and four normal thyroid tissues (see Supplementary Material, Table S1) by hybridizing the RNA extracted from these samples to the Human LncRNA Microarray Version 3.0 (Arraystar, Rockville, MD, USA). After hybridization and normalization of the raw data, we obtained through bioinformatic analysis a list of 669 and 2470 lncRNAs that resulted up- and down-regulated, respectively, in the PTC tissues analysed compared to normal thyroid samples with fold change >2 and *p*-value < 0.05. The complete list of up- and down-regulated lncRNAs is provided in Supplementary Material (Table S2). A representative partial list of deregulated lncRNAs is shown in Table 1.

**Figure 1:**
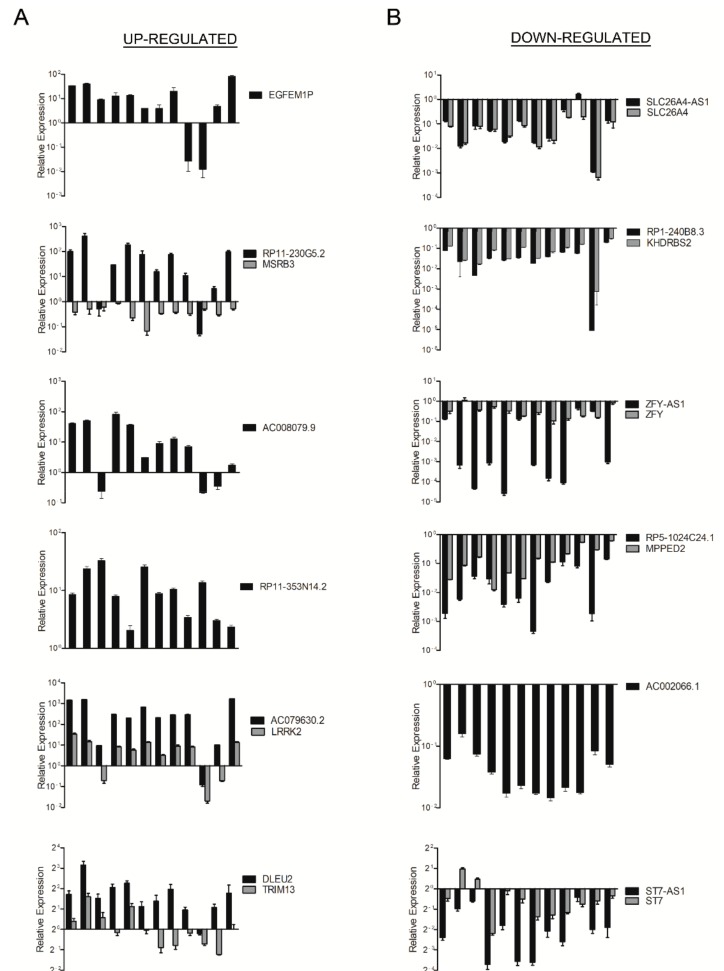
Analysis of lncRNA and gene expression in papillary thyroid carcinoma (PTC). Total RNA extracted from 12 PTC and four normal thyroid samples was hybridized to a lncRNA microarray. qRT-PCR analysis was performed to evaluate the expression of up-regulated (**A**) and down-regulated (**B**) lncRNAs and their associated genes. Results are reported as relative expression ± SEM compared to the mean of four normal thyroid samples set equal to 1.

To validate the results obtained by the microarray analysis, we selected six up- (*EGFEM1P*, *RP11-230G5.2*, *AC008079.9*, *RP11-353N14.2*, *AC079630.2* and *DLEU2*) and six down-regulated lncRNAs (*SLC26A4-AS1*, *RP1-240B8.3*, *ZFY-AS1*, *RP5-1024C24.1*, *AC002066.1* and *ST7-AS1*) with respect to normal thyroid tissues. Then, we evaluated their expression by qRT-PCR in the same PTC and normal thyroid samples previously used for the lncRNA microarray thus confirming the results obtained. In fact, as shown in Figure 1A,B, we found that the expression of *EGFEM1P*, *RP11-230G5.2*, *AC008079.9*, *RP11-353N14.2*, *AC079630.2* and *DLEU2* was up-regulated in PTC, while the expression of *SLC26A4-AS1*, *RP1-240B8.3*, *ZFY-AS1*, *RP5-1024C24.1*, *AC002066.1* and *ST7-AS1* resulted down-regulated. Moreover, we selected the lncRNAs *RP11-230G5.2*, *DLEU2* and *SCL26A4-AS1* and analysed their expression levels in additional 12 PTC comparing them to the mean of three normal thyroid tissues (three out of the four normal thyroid samples used for microarray hybridization and validation, see Materials and Methods section). By qRT-PCR, we observed also in this case an up-regulation of *RP11-230G5.2* and *DLEU2*, and a down-regulation of *SCL26A4-AS1*, thus supporting the lncRNA microarray analysis (see Supplementary Material, Figure S1).

As shown in Table S2, the lncRNAs identified were classified on the basis of their genomic orientation with respect to their neighbouring genes in exon-sense overlapping, intron-sense overlapping, intronic antisense, natural antisense, bidirectional and intergenic [11,12]. Since several studies have recently demonstrated that lncRNAs frequently act through the modulation of their associated neighbouring gene expression [21,22,23,24,25,26], we focused our attention on the antisense class of lncRNAs for further investigations. By the lncRNA microarray analysis, we observed the following association between lncRNAs and related genes: *RP11-230G5.2*, antisense with respect to the *MSRB3* gene; *AC079630.2*, intergenic with respect to the *LRRK2* gene; *DLEU2*, antisense with respect to the *TRIM13* gene; *SLC26A4-AS1*, antisense with respect to the *SLC26A4* gene; *RP1-240B8.3* antisense with respect to the *KHDRBS2* gene; *ZFY-AS1*, antisense with respect to the *ZFY* gene; *RP5-1024C24.1*, antisense with respect the *MPPED2* gene; *ST7-AS1*, antisense with respect to the *ST7* gene (Table 1 and Table S2). Therefore, we evaluated, by qRT-PCR, the expression of the selected lncRNA-associated genes in the same PTC and normal thyroid microarray samples. Interestingly, we found a positive association between the lncRNAs and their related gene expression in six out of eight lncRNA/neighbouring-gene pairs analysed (Figure 1A,B). Conversely, a negative association was observed between *RP11-230G5.2* and *MSRB3* expression, and no association was observed between *DLEU2* and *TRIM13* expression (Figure 1A).

### 2.2. Analysis of RP5-1024C24.1 and MPPED2 Expression in Neoplastic Thyroid Diseases

To characterize the role of lncRNAs in thyroid carcinogenesis, we decided to focus our study on the lncRNA *RP5-1024C24.1*, located on chromosome 11 in antisense position with respect to the *MPPED2* gene encoding a metallophosphoesterase protein. We made this choice since both of them were drastically down-regulated in all the PTC samples analysed by the lncRNA microarray, and then by qRT-PCR. Moreover, *MPPED2* gene has been already reported to play an important anti-oncogenic role in oral squamous cell carcinoma [27], cervical cancer [28] and neuroblastoma [29]. Therefore, we decided to further investigate the functional role of the lncRNA *RP5-1024C24.1* and its associated *MPPED2* gene in human thyroid carcinomas and their possible relationship in thyroid cancer.

**Figure 2:**
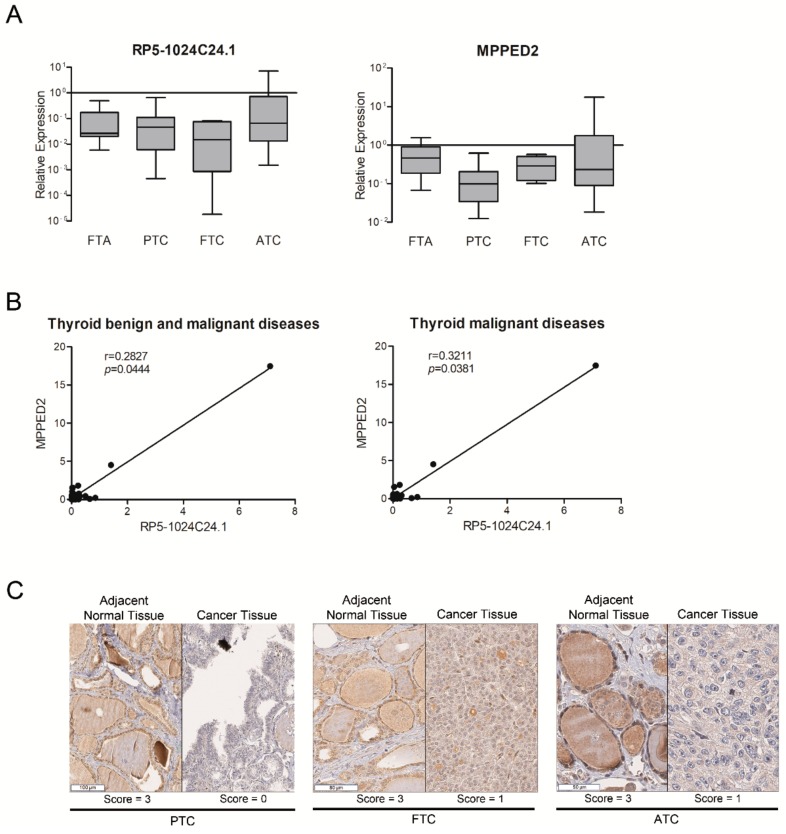
Analysis of *RP5-1024C24.1* and *MPPED2* expression in thyroid neoplastic diseases. (**A**) qRT-PCR analysis performed on FTA (*n* = 9), PTC (*n* = 12 previously used for lncRNA microarray hybridization and *n* = 12 additional samples), FTC (*n* = 6), ATC (*n* = 12) to evaluate the expression of *RP5-1024C24.1* (left panel) and *MPPED2* (right panel). Results are reported as relative expression compared to the mean of normal thyroid samples, set equal to 1 (box and whiskers, min to max). (**B**) Correlation scatterplot (Spearman test) of *RP5-1024C24.1* and *MPPED2* mRNA levels (relative expression) in thyroid benign and malignant diseases (FTA, PTC, FTC, ATC) (left panel). Correlation scatterplot (Spearman test) of *RP5-1024C24.1* and *MPPED2* mRNA levels (relative expression) in thyroid carcinomas (PTC, FTC, ATC) (right panel). (**C**) Representative immunohistochemical staining of MPPED2 protein in thyroid carcinoma samples (PTC, FTC, ATC) and in their corresponding adjacent normal tissue. The MPPED2 signal is strong in normal thyroid tissues (Score = 3) and mild (Score = 1) or absent (Score = 0) in thyroid carcinoma tissues (magnification 40×).

To this aim, we analysed by qRT-PCR the expression levels of both *RP5-1024C24.1* and *MPPED2* in a set of nine FTA samples, additional 12 PTC samples, six FTC and 12 ATC and compared them to the mean of three normal thyroid tissues (three out of the four normal thyroid samples used for microarray hybridization and validation, see Materials and Methods section). As shown in Figure 2A, we observed a reduced expression of both genes with respect to normal thyroid tissues in all the thyroid neoplastic histotypes analysed, including the FTA samples. Moreover, we found a significant (*p* = 0.0444) positive correlation between their expression in the whole neoplasm set analysed, suggesting that both *RP5-1024C24.1* and *MPPED2* are co-regulated during the process of thyroid carcinogenesis (Figure 2B, left panel). The positive correlation between *RP5-1024C24.1* and *MPPED2* expression was still significant when we consider only the thyroid malignant samples (PTC, FTC, ATC) (*p* = 0.0381) (Figure 2B, right panel). These results were then confirmed at the protein level by immunohistochemical analysis using an anti-MPPED2 antibody. In fact, reduced MPPED2 protein levels were found in 37% of FTA (7 out of 19 cases), 79% of PTC (116 out of 147 cases), 77% of FTC (23 out of 30 cases), 86% of PDC (32 out of 37 cases) and 53% of ATC (8 out of 15 cases) in comparison to normal matched thyroid tissue (Table 2).

Representative results are shown in Figure 2C. The signal corresponding to MPPED2 was strong (Score = 3) with a granular cytoplasmic expression in the normal tissues. Conversely, in the paired PTC, FTC and ATC samples shown in the same figure, the signal corresponding to MPPED2 was completely absent (Score = 0) or mild and non-granular (Score = 1) (see Materials and Methods section). Statistical analyses of MPPED2 expression in thyroid carcinoma samples compared to their adjacent normal tissue reveal significant differences between tumours and their corresponding normal tissue in the following categories: whole cohort (*p* < 0.001), PTC (*p* < 0.001), FTC (*p* < 0.001), PDC (*p* < 0.001), ATC (*p* = 0.003) (Table 2). We did not observe any significant differences of MPPED2 expression levels between the different histological groups. Moreover, as far as the association of differential MPPED2 expression between tumour and normal matched samples with clinico-pathological information is concerned, we did not find any significant correlation with the TNM stage, morphological tumour characteristics and the mutational status of specific thyroid cancer-related genes (data not shown).

### 2.3. Down-Regulation of RP5-1024C24.1 Expression Contributes to Thyroid Carcinogenesis by Affecting the PTEN/Akt Pathway

Subsequently, to better define the role of *RP5-1024C24.1* in thyroid carcinogenesis, we modulated its expression in thyroid carcinoma cell lines. To achieve this aim, we first analysed the expression of this gene by qRT-PCR in a panel of thyroid carcinoma cell lines, including TPC-1 and B-CPAP (PTC-derived cell lines), WRO (FTC-derived cell line) and FB-1 and FRO (ATC-derived cell lines). As shown in Figure S2, the expression of *RP5-1024C24.1* was much lower in all the cell lines analysed in comparison with three normal thyroid samples used as control (see Materials and Methods section).

**Figure 3:**
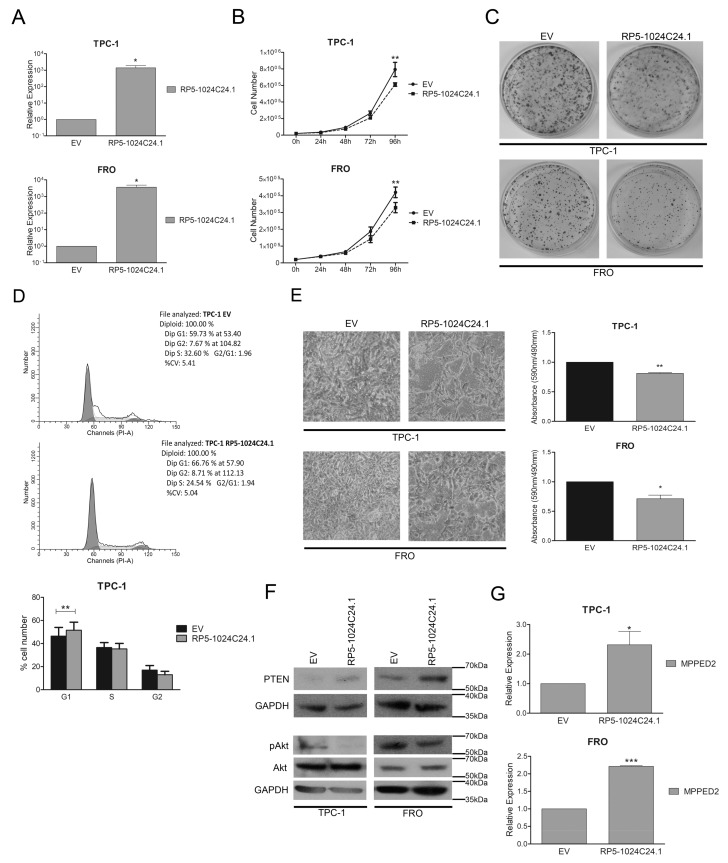
*RP5-1024C24.1* reduces cell migration and proliferation of thyroid carcinoma cell lines. (**A**) qRT-PCR analysis performed on TPC-1 and FRO cell lines stably carrying *RP5-1024C24.1* or the corresponding empty vector (*EV*). Results were obtained from four independent experiments. Data were compared to EV, set equal to 1, and reported as relative expression ± SEM. *t*-test; * *p* < 0.05. (**B**) Cell growth analysis of TPC-1 and FRO stably carrying *RP5-1024C24*.1 or *EV*. Cell number was evaluated at 24 h, 48 h, 72 h and 96 h after seeding. Values were obtained from three independent experiments performed in duplicate. Data were reported as mean ± SEM. 2-way Anova-test followed by Bonferroni post-test; ** *p* < 0.01. (**C**) Representative colony assay performed on TPC-1 and FRO cell lines stably carrying *RP5-1024C24.1* or *EV*. (**D**) Representative cell cycle analysis of TPC-1 cell lines stably carrying *RP5-1024C24.1* or *EV*. Cell number was reported on the *y*-axis while the percentage of propidium iodide (PI) incorporated was reported on the *x*-axis (upper panel). Values shown in the lower panel were obtained from five independent experiments. *t*-test; ** *p* < 0.01 compared to *EV* cells. (**E**) Representative acquisition of migration assays performed on TPC-1 and FRO stably carrying *RP5-1024C24.1* or *EV* (magnification 40×) (left panel). Data obtained from three (TPC-1) or four (FRO) independent experiments are shown in the right panel. Values were reported as mean value ± SEM and compared to the *EV*, set equal to 1. *t*-test; * *p* < 0.05; ** p < 0.01. (**F**) Immunoblot analysis performed on TPC-1 and FRO cell lines stably carrying *RP5-1024C24.*1 or *EV* to analyze the protein level of PTEN, Akt and pAkt. GAPDH was used to normalize the amount of loaded protein. (**G**) *MPPED2* expression evaluated by qRT-PCR in TPC-1 and FRO stably expressing *RP5-1024C24.1*. Data were obtained from five (TPC-1) or three (FRO) independent experiments. Values were reported as relative expression ± SEM and were compared to the *EV*, set equal to 1. *t*-test; * *p* < 0.05; *** *p* < 0.001.

Then, we restored the expression of *RP5-1024C24.1* in TPC-1 and FRO cell lines by transfecting them with a vector expressing the lncRNA and selected the transfected cells in a G418-containing medium. Next, we confirmed by qRT-PCR the restoration of *RP5-1024C24.1* expression in the selected cells (Figure 3A and Figure S3A) and evaluated its effects on cell proliferation. As shown in Figure 3B, both TPC-1 and FRO cells expressing *RP5-1024C24.1* displayed a significant reduction in the cell growth rate compared to the respective empty vector transfected cells. Consistently, cell colony-forming assays evidenced that *RP5-1024C24.1* reduces the number of colonies compared to the control cells (Figure 3C), thus confirming that *RP5-1024C24.1* is able to negatively modulate the cell proliferation of both PTC and ATC cells.

Cell cycle analysis performed by flow cytometry showed that *RP5-1024C24.1* increased the number of TPC-1 cells in the G1 phase compared to the empty vector (TPC-1-*EV*, G1: 59.73%; TPC-1-*RP5-1024C24.1*, G1: 66.76%; *p* = 0.0092) (Figure 3D). Moreover, to verify whether the restoration of *RP5-1024C24.1* has an effect on other cancer-associated processes as well, we analysed the cell migration rate of TPC-1 and FRO stably expressing *RP5-1024C24.1* by transwell assays after inhibiting cell proliferation with mytomicin C. As shown in Figure 3E, we found that the restoration of the lncRNA expression was also able to reduce the migration ability of the selected thyroid carcinoma cell lines.

Next, to characterize the molecular mechanisms by which the lncRNA *RP5-1024C24.1* may act, we evaluated the expression of PTEN, a well-known oncosuppressor protein [30,31], in TPC-1 and FRO cells stably expressing the lncRNA. By western blot analysis, we found that *RP5-1024C24.1* is able to increase PTEN levels (Figure 3F, upper panel). In addition, we analysed the effect of the PTEN induction on its down-stream effector Akt [32,33] and found that cells expressing *RP5-1024C24.1* showed a reduced Akt phosphorylation on Ser473 (Figure 3F, lower panel) resulting in the reduction of its activation [34].

Therefore, these results indicate that *RP5-1024C24.1* can affect cell proliferation and migration through a mechanism that involves the modulation of the PTEN/Akt pathway, further supporting the anti-oncogenic role played by *RP5-1024C24.1* in thyroid carcinogenesis.

### 2.4. MPPED2 Is Induced by RP5-1024C24.1 and Negatively Modulates Cell Proliferation and Migration of Thyroid Carcinoma Cell Lines

To investigate whether *RP5-1024C24.1* modulates the expression of its-associated *MPPED2* gene, we analysed the expression of *MPPED2* by qRT-PCR in TPC-1 and FRO stably expressing *RP5-1024C24.1*. Interestingly, we found that *RP5-1024C24.1* is able to increase the expression of *MPPED2* in both cell lines with respect to the empty vector transfected cells, thus suggesting that the lncRNA may act also through the modulation of the *MPPED2* expression (Figure 3G).

**Figure 4:**
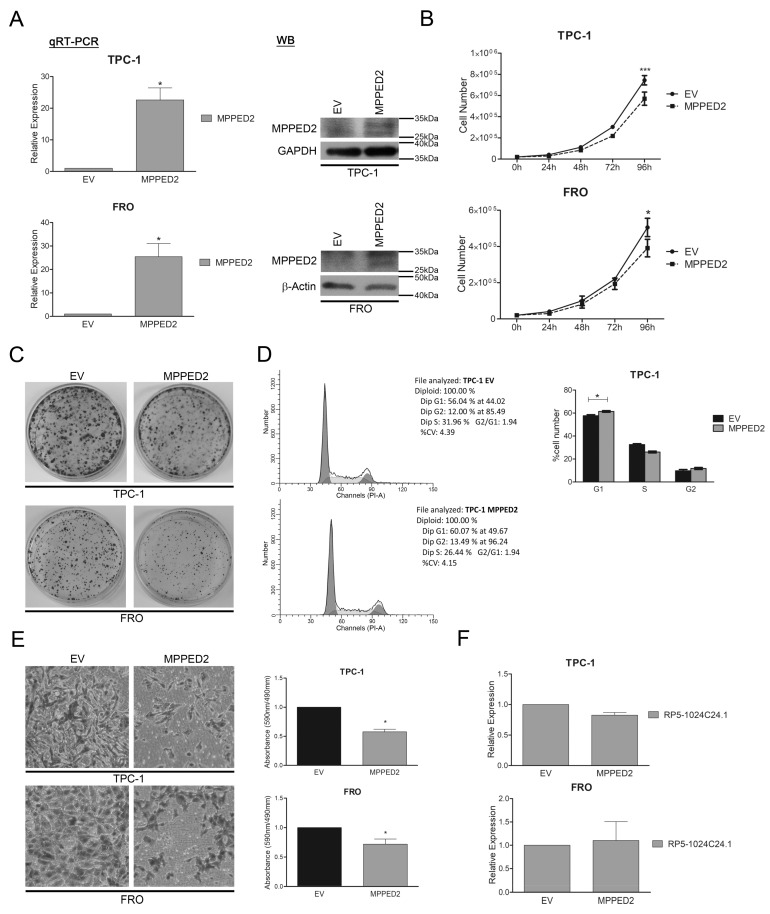
MPPED2 negatively modulates cell proliferation and migration of thyroid carcinoma cell lines. (**A**) qRT-PCR analysis performed on TPC-1 and FRO cell lines stably carrying *MPPED2* or the corresponding empty vector (*EV*). Values were reported as relative expression ± SEM and were compared to *EV*, set equal to 1. *t*-test; * *p* < 0.05 (left panel). Immunoblot analysis confirming the expression of MPPED2. GAPDH and β-Actin were used to normalize the amount of loaded protein (right panel). (**B**) Cell growth analysis of TPC-1 and FRO stably carrying *MPPED2* or *EV*. Cell number was evaluated at 24 h, 48 h, 72 h and 96 h after seeding. Values were obtained from three independent experiments performed in duplicate and data were reported as mean ± SEM. 2-way Anova-test followed by Bonferroni post-test; * p < 0.05; *** *p* < 0.001. (**C**) Representative colony assay performed on TPC-1 and FRO cell lines stably carrying *MPPED2* or *EV*. (**D**) Representative cell cycle analysis of TPC-1-*MPPED2* and TPC-1-*EV* cells. Cell number was reported on the *y*-axis while the percentage of propidium iodide (PI) incorporated was reported on the *x*-axis (left panel). Values shown in the right panel were obtained from three independent experiments. *t*-test; * *p* < 0.05 compared to *EV*. (**E**) Representative acquisition of migration assays performed on *MPPED2* or *EV* transfected TPC-1 and FRO cells (magnification 40×) (left panel). Values obtained from three (TPC-1) or four (FRO) independent experiments were reported as mean ± SEM and compared to the *EV*, set equal to 1 (right panel). *t*-test; * *p* < 0.05. (**F**) qRT-PCR analysis to evaluate the expression of *RP5-1024C24.1* after *MPPED2* transfection. Data obtained from three (TPC-1) or five (FRO) independent experiments were reported as relative expression ± SEM and were compared to the *EV*, set equal to 1. *t*-test; *p* = *ns*.

Next, to characterize the role that this gene plays in thyroid carcinogenesis, we analysed by qRT-PCR the expression of *MPPED2* in a panel of thyroid carcinoma cell lines (Figure S2) and then we stably restored its expression in TPC-1 and FRO cell lines. After G418 selection, we confirmed the increased expression of MPPED2 in the transfected cells (Figure 4A and Figure S3B) and evaluated its functional effects. As displayed in Figure 4B,C, the growth curve and colony formation assays showed that both the *MPPED2*-transfected TPC-1 and FRO cells have a lower proliferation rate than cells carrying the empty vector. Moreover, cell cycle progression analysis performed on TPC-1 cells through flow cytometry confirmed the negative effect of *MPPED2* on cell proliferation by showing an increased percentage of cells in the G1 phase when compared to cells carrying the empty vector (TPC-1-*EV*, G1: 56.04%; TPC-1-*MPPED2*, G1: 60.07%; *p* = 0.0367) (Figure 4D).

In addition, to evaluate the effects of *MPPED2* on cancer progression, we performed transwell assays on *MPPED2*-expressing TPC-1 and FRO cells treated with mytomicin C. Interestingly, as shown in Figure 4E, we found a significant reduction of migrating cells both in *MPPED2*-TPC-1 and FRO cells, thus demonstrating a role of *MPPED2* in inhibiting also cellular migration.

Noteworthy, through qRT-PCR, we observed no modulation of the *RP5-1024C24.1* expression mediated by *MPPED2*, thus indicating that *RP5-1024C24.1* is able to modulate *MPPED2*, but not vice versa (Figure 4F).

## 3. Discussion

The aim of our study was to investigate the role of lncRNAs in thyroid carcinogenesis by evaluating the expression profile of 12 PTC compared to four normal thyroid samples through a human lncRNA microarray approach. This analysis identified a relevant number of lncRNAs deregulated in PTC compared to normal thyroid tissues. The microarray results were confirmed by evaluating the expression of six up- and six down-regulated lncRNAs by qRT-PCR. Subsequently, we focused our attention on the *RP5-1024C24.1* lncRNA, that was drastically down-regulated in all PTC samples analysed with respect to normal thyroid tissues. However, we are currently planning to examine in depth the role of other deregulated lncRNAs in thyroid carcinogenesis.

The next step of our investigation was to evaluate the expression of *RP5-1024C24.1* in thyroid neoplastic samples of different malignant degrees. Interestingly, we observed that the expression levels of this lncRNA are decreased in both differentiated and undifferentiated thyroid carcinomas. Surprisingly, *RP5-1024C24.1* reduction was also observed in benign FTA suggesting a key role of *RP5-1024C24.1* down-regulation even in the early phases of thyroid cell neoplastic transformation.

Bioinformatic analyses (Materials and Methods section) revealed that *RP5-1024C24.1* is located on the genome in an antisense position with respect to *MPPED2.* This gene codes for an enzyme belonging to the III class-family of phosphoesterase in mammals and is highly expressed in foetal brain. Recent studies have demonstrated that the expression of *MPPED2* is drastically down-regulated in several malignant neoplasias originating from different tissues. Moreover, its restoration in cancer cell lines induces apoptosis and negatively modulates cell proliferation [27,28,29], thus proposing *MPPED2* as a potential candidate tumour suppressor gene. Consequently, we evaluated the expression of the *MPPED2* gene by qRT-PCR in the same set of thyroid neoplastic samples analysed for *RP5-1024C24.1* expression: a reduced expression of this gene was observed in the different histotypes analysed. In addition, we observed a significant positive correlation between *MPPED2* and *RP5-1024C24.1* expression in the thyroid neoplastic samples analysed (*p* = 0.0444).

Noteworthy, immunohistochemical analysis performed on a large number of paraffin-embedded thyroid neoplastic tissues and their adjacent normal thyroid tissue confirmed the results obtained by qRT-PCR. In fact, we observed that MPPED2 levels were moderately decreased in FTA, and strongly decreased in FTC, PTC and PDC. Surprisingly, while the expression of MPPED2 was markedly reduced in 86% of PDC samples, in the ATC set only 53% of cases showed reduced expression of MPPED2. Additionally, by analysing each thyroid carcinoma histotype, we found significant differences of MPPED2 expression between neoplastic and normal tissue samples.

Functional studies were performed to define the role of *RP5-1024C24.1* and *MPPED2* down-regulation in thyroid carcinogenesis. Accordingly, we stably restored the expression of *RP5-1024C24.1* in two thyroid carcinoma cell lines by transfecting them with a vector expressing the lncRNA sequence. We observed that *RP5-1024C24.1* was able to reduce the cell proliferation and migration rate, thus indicating that the deregulation of this lncRNA might play a causative role in the modulation of the biological processes leading to thyroid carcinoma development.

Interestingly, in order to clarify the molecular mechanisms by which *RP5-1024C24.1* is able to affect cell growth and migration in thyroid carcinoma cells, we found that the restoration of *RP5-1024C24.1* is able to increase PTEN protein levels and to reduce Akt-Ser473 phosphorylation, thus suggesting that the modulation of this pathway could account for the effects of *RP5-1024C24.1* on cell proliferation and migration [30,31,32,33,34].

However, since we demonstrated that also *MPPED2* restoration is able to inhibit cell proliferation and migration in TPC-1 and FRO cell lines, we suggest that the functional effects of *RP5-1024C24.1* could also be due to the up-regulation of the *MPPED2* gene expression. Interestingly, no *RP5-1024C24.1* modulation was observed in *MPPED2*-expressing cells indicating that the regulation is unidirectional.

As far as the mechanisms by which *RP5-1024C24.1* is able to positively regulate *MPPED2* are concerned, they still need to be clarified, although several pieces of evidence suggest that lncRNAs are able to modulate the expression of their associated genes through epigenetic regulations [21,22,23,24,25,26]. Consistently, Shen and colleagues have recently indicated miR-448 as a negative regulator of *MPPED2* [27]. Therefore, we have analysed the expression of this miRNA in our thyroid carcinoma cell systems stably expressing *RP5-1024C24.1*. Interestingly, our preliminary data have shown a reduction of the miR-448 expression following the restoration of the lncRNA expression (data not shown), thus suggesting that one of the mechanisms by which *RP5-1024C24.1* might positively regulate *MPPED2* expression could be through the negative modulation of the miR-448.

In conclusion, the results presented here indicate that the down-regulated *RP5-1024C24.1* and its associated-gene *MPPED2*, could represent novel tumour suppressor genes with a considerable role in thyroid cell neoplastic transformation and progression.

## 4. Materials and Methods

### 4.1. Human Thyroid Samples

The whole set of human thyroid carcinoma specimens used was provided by the Service d’Anatomie et Cytologie Pathologiques, Centre de Biologie Sud, Groupement Hospitalier Lyon Sud, Pierre Bénite, France. The histotype and TNM characteristics of PTC samples used for lncRNA microarray analysis are reported in Table S1. The activity of biological samples conservation was declared under the number DC-2011-1437 to the ministry of Research, to the committee of people’s protection of south-east IV and to the Health Regional Agency. The activity of biological material cession was agreed upon by the ministry of Health under the number AC-2013-1867.

### 4.2. Long Non-Coding RNA Microarray Analysis

Total RNA extracted from 12 PTC samples and four normal thyroid tissues was hybridized to the Human LncRNA Microarray Version 3.0 of the Arraystar company (Rockville, MD, USA). This system is based on probes able to recognize specific exons or splice junction of each lncRNA. The expression analysis was performed by comparing the average of the expression levels observed in 12 PTC samples with the average of the expression levels observed in four normal thyroid tissues. Bioinformatic analyses were performed by the Arraystar company based on the following databases: Refseq, UCSC, GENCODE, RNAdb, NRED, UCR, lincRNA catalogs [35,36] (https://www.arraystar.com/human-lncrna-expression-microarray-v4-0/) (Table S2).

### 4.3. Cell Lines and Transfection

TPC-1 and FRO cell lines were grown in DMEM (Sigma-Aldrich, St. Louis, MO, USA) supplemented with 10% foetal bovine serum (Euroclone, Milan, Italy), 1% l-glutamine, 1% penicillin/streptomycin (Sigma-Aldrich) and were maintained at 37 °C under 5% CO_2_ atmosphere.

The authenticity of cell lines has been confirmed through short tandem repeat (STR) profiling.

TPC-1 cells were transfected using Fugene HD reagent (Promega, Fitchburg, WI, USA) while FRO cells were transfected using the Lipofectamine 2000 reagent (Life Technologies, Grand Island, NY, USA), according to the manufacturer’s instructions. For stably-expressing cell lines, TPC-1 and FRO were selected by using 1000 µg/mL and 1200 µg/mL of G418 (Life Technologies), respectively.

For cell count number assays, 2 × 10^4^ TPC-1 and FRO stable clones were seeded in a six well plate in duplicate and counted after 24 h, 48 h, 72 h, 96 h using a Burker chamber.

### 4.4. Plasmids

The *RP5-1024C24.1* expression vector was obtained by cloning the lncRNA sequence in the pCMV6-AC-GFP vector (Origene Technologies, Rockville, MD, USA) using the HindIII and XhoI restriction sites. The pCMV6-MPPED2-DDK-myc expression vector encoding human MPPED2 (NM_00145399.1) fused to the myc/DDK epitope in the C-terminal region was purchased from Origene Technologies (RC227201).

### 4.5. RNA Extraction and qRT-PCR

Total RNA was extracted from cell lines and tissues by using Trizol reagent (Life Technologies), according to the manufacturer’s instructions. 1 μg of total RNA was used to obtain a double strand cDNA with the QuantiTect Reverse Transcription Kit (Qiagen, Hilden, Germany). qRT-PCR was carried out in a 96 well plate with the CFX 96 thermocycler (Bio-Rad, Hercules, CA, USA) using 20 ng of each cDNA and SYBR Green (Bio-Rad). *G6PD* was used as reference gene for qRT-PCR performed on thyroid neoplasias, while *β-Actin* was used for thyroid carcinoma cell lines. Primers sequences are listed in Table S3.

Relative expression values were calculated according to the 2^−ΔΔCt^ formula as previously described [37].

As far as qRT-PCR analyses are concerned, we used four normal thyroid tissue samples for the validation of the microarray results (lncRNA and associated gene expression in PTC). However, we used only three out of four normal thyroid tissues for further analyses (expression analyses in additional set of thyroid neoplasms and cell lines) since the RNA of one out of the four normal samples ran out.

### 4.6. Protein Extraction and Western Blot Analysis

Total protein extracts were obtained using the lysis buffer (120 mM NaCl, 20 mM Tris-HCl pH 7.5, 2% Nonidet P40) completed with a mix of proteases and phosphatases inhibitors. 80 μg of extracted proteins were separated by SDS-PAGE and then transferred onto PVDF membranes (Merck Millipore, Darmstadt, Germany). Membranes were blocked with BSA or 5% not-fat milk and then incubated with the following antibodies: anti-myc tag (ab9132, Abcam, Cambridge, UK), anti-MPPED2 (H00000744-D01P, Abnova, Taipei City, Taiwan), anti-PTEN (ab32199, Abcam), anti-pospho-Akt (Ser473) (#4051, Cell Signaling, Danvers, MA, USA), anti-Akt (#92725, Cell Signaling). To normalize the amount of protein loaded, the membranes were incubated with anti-GAPDH (Santa Cruz Biotechnology Inc., Santa Cruz, CA, USA) and anti-β-Actin protein (Sigma-Aldrich). Filters were then incubated with horseradish peroxidase-conjugated secondary antibody (1:3000) for 1 h at room temperature and the signals were detected by western blotting detection system (ECL).

### 4.7. Cell Migration Assays

Transwell motility assays were performed as previously described [38]. Briefly, cells were first treated with mytomicin C (Sigma-Aldrich) at a final concentration of 0.01 mg/mL for 3 h. Then, 3 × 10^4^ TPC-1 and FRO cells were seeded both in the transwell for migration and in a 96 well plate in triplicate or quadruplicate to normalize the number of cells used for each cell line. The cell titer and the crystal violet de-stained with PBS-0.1% SDS solution were read at 490 nm and 590 nm, respectively, in a microplate reader (LX 800, Universal Microplate Reader, BioTek, Winooski, VT, USA). Results were obtained by normalizing the crystal violet values to cell titer ones.

### 4.8. Immunohistochemical Analysis

In the IHC analysis, the following set of thyroid neoplasias and the corresponding adjacent normal thyroid tissue was included: FTA, *n* = 19; PTC, *n* = 147 (classical variant, *n* = 74; follicular variant, *n* = 73); FTC, *n* = 30; PDC, *n* = 37; ATC, *n* = 15.

The original Hematoxylin–Eosin–Saffron (HES) stained slides were reviewed. For the PTC cases, the most representative areas of normal and tumoural tissues were circled. Up to two formalin-fixed paraffin-embedded (FFPE) blocks were selected to be sampled in a tissue microarray (TMA). One 0.6 mm normal tissue core and three 0.6 mm tumoural tissue cores were collected and aligned in two TMA blocks, using the tissue arrayer Minicore^®^ 3 (Alphelys, Plaisir, France). The blocks were sectioned at 3 µm. An HES stain of the TMAs was performed to assess the representativeness of the cores. The ATC and FTA cases were processed as whole slides following the same immunohistochemical protocol as the TMAs.

Immunohistochemical staining was performed on a Benchmark Ultra automated staining platform (Ventana, Tucson, AZ, USA) using a rabbit polyclonal MPPED2 antibody (Abnova), with a 1:60 dilution and an UltraView DAB detection kit (Ventana). A hematoxylin counterstain followed.

The level of staining was evaluated in the normal and tumoural tissues following a qualitative scoring method. The expected staining pattern was “cytoplasmic granular”, conformingly to what was observed in the positive controls (normal duodenal epithelium). The absence of staining was scored 0, a mild non-granular staining was scored 1, a granular moderate staining was scored 2 and a granular intense staining was scored 3.

The difference of MPPED2 expression between non tumoural and tumoural tissue, in the whole cohort and in each histologic subtype, was analysed using the paired sample *t*-test.

### 4.9. Flow Cytometry Analysis

For cell cycle analyses, cells were processed as previously described [39]. Briefly, cells were trypsinized and, after washing in PBS, fixed in 70% ethanol. After a centrifugation at 1200 rpm for 10 min at 4 °C, cells were treated with 50 µg/mL propidium iodide and 25 µg/mL ribonuclease A in PBS for 20 min at RT safe of light. For each measurement 10,000 events were analysed using a FACScanto II flow cytometer (Becton Dickinson, San Jose, CA, USA) and then cell cycle data were analysed with the ModFit LT 2.0 software (Verity Software House, Topsham, ME, USA) in a semiautomatic analysis procedure. The ModFit algorithm was finally used to analyse the files obtained, calculating the percentage of cells in each cell cycle phase.

### 4.10. Statistical Analysis

GraphPad Prism software was used for statistical analyses. *t*-test and Anova tests were used to evaluate the statistical significance of the obtained data, while gene expression correlation was evaluated through non-parametric Spearman’s Rank correlation coefficient. When Anova test was significant (*p* < 0.05), we determined the differences between groups using Bonferroni post-test. In all the experiments, the significance was assessed for *p* < 0.05. Data are reported as mean values ± standard error of mean (SEM).

## 5. Conclusions

In this study, we identified several lncRNAs whose expression is deregulated in PTC compared to normal thyroid samples and, among them, we focused on *RP5-1024C24.1* and on its associated-antisense gene *MPPED2* for further investigation. We report that both genes are down-regulated in thyroid neoplasias. Moreover, the restoration of their expression in thyroid cancer cell lines reduces cell proliferation and migration, thus suggesting a tumour suppressor role for *RP5-1024C24.1* and *MPPED2* in the development of thyroid neoplasias.

## Figures and Tables

**Table 1:** Representative list of lncRNAs deregulated in papillary thyroid carcinomas (PTC) vs. normal thyroid tissues (NT) ^1^.

**Up-Regulated LncRNAs (PTC vs. NT)**							
**Gene Symbol**	**Seqname**	**Fold Change**	***p*** **-Value**	**Chr.**	**Strand**	**Relationship**	**Associated Gene**
*XLOC_010052*	TCONS_00020760	44.796062	0.011679098	chr12	−	intergenic	
*EGFEM1P*	ENST00000488647	29.21525	0.003039263	chr3	+	intergenic	
*RP11-230G5.2*	ENST00000538294	16.403324	0.003381364	chr12	−	natural antisense	*MSRB3*
*AC008079.9*	ENST00000434390	15.255612	0.017524553	chr22	−	intergenic	
*RP11-353N14.2*	ENST00000576963	14.603789	6.1 × 10^−^^5^	chr17	+	intergenic	
*AC079630.2*	ENST00000457989	12.705946	0.00535464	chr12	+	intergenic	*LRRK2*
*CTC-255N20.1*	ENST00000504297	6.02792	0.001935031	chr5	−	bidirectional	*STK32A*
*AC003102.3*	ENST00000453562	5.626362	7.34 × 10^−^^4^	chr17	−	natural antisense	*RUNDC3A*
*DLEU2*	uc001vdo.1	4.361354	9.4 × 10^−^^4^	chr13	−	natural antisense	*TRIM13*
*RP11-4C20.4*	ENST00000433110	3.8502407	0.035742607	chr10	+	intron sense-overlapping	*PTPRE*
**Down-Regulated LncRNAs (PTC vs. NT)**							
**Gene Symbol**	**Seqname**	**Fold Change**	***p*** **-Value**	**Chr.**	**Strand**	**Relationship**	**Associated Gene**
*CTB-85P21.2*	ENST00000566630	72.72284	6.38 × 10^−^^7^	chr5	+	intergenic	
*SLC26A4-AS1*	NR_028137	27.245485	0.006468582	chr7	−	natural antisense	*SLC26A4*
*RP11-317P15.5*	ENST00000570153	20.019192	0.007198248	chr1	−	intergenic	
*RP1-240B8.3*	ENST00000511849	17.767982	0.001431982	chr6	−	exon sense-overlapping	*KHDRBS2*
*ZFY-AS1*	ENST00000417305	14.560697	0.017612867	chrY	−	natural antisense	*ZFY*
*LA16c-329F2.1*	ENST00000570022	12.498036	0.031231718	chr16	−	intronic antisense	*MAPK8IP3*
*RP5-1024C24.1*	ENST00000531002	10.285704	7.4 × 10^−^^6^	chr11	+	intronic antisense	*MPPED2*
*AC002066.1*	ENST00000439070	9.467349	8.22 × 10^−^^4^	chr7	−	intergenic	
*ST7-AS1*	NR_002330	8.9516325	1.38 × 10^−^^4^	chr7	−	natural antisense	*ST7*
*RP11-121G22.3*	ENST00000553197	8.723473	5.89 × 10^−^^6^	chr12	+	intronic antisense	*PPFIA2*

^1^ For each lncRNA we report the gene symbol, accession number, the fold change expression compared to normal thyroid tissues, the *p*-value of the analysis, the chromosome and the DNA strand in which they are located, their classification and the name of their associated gene on the genome.

**Table 2:** Expression of MPPED2 protein levels in thyroid neoplasias analysed by immunohistochemistry.

Histotype (*n*)	Reduced Expression ^1^ (*n*, %)	Whole Cohort
FTA ^2^ (*n* = 19)	*n* = 7 (37%)	*p* < 0.001
PTC ^3^ (*n* = 147)	*n* = 116 (79%)	*p* < 0.001
FTC ^4^ (*n* = 30)	*n* = 23 (77%)	*p* < 0.001
PDC ^5^ (*n* = 37)	*n* = 32 (86%)	*p* < 0.001
ATC ^6^ (*n* = 15)	*n* = 8 (53%)	*p* = 0.003
Whole cohort *p* < 0.001		

^1^ MPPED2 protein expression in thyroid carcinoma vs. the adjacent corresponding normal thyroid tissue; ^2^ FTA, follicular thyroid adenoma; ^3^ PTC, papillary thyroid carcinoma; ^4^ FTC, follicular thyroid carcinoma; ^5^ PDC, poorly differentiated carcinoma; ^6^ ATC, anaplastic thyroid carcinoma.

## Supplementary Materials

The following are available online at http://www.mdpi.com/2072-6694/10/5/146/s1, Figure S1: LncRNA expression analysis in additional PTC samples. qRT-PCR analysis to evaluate the expression of RP11-230G5.2, DLEU2 and SLC26A4-AS1 in additional PTC samples. Results are reported as relative expression ± SEM compared to the mean of three normal thyroid samples set equal to 1; Figure S2: Expression analysis of *RP5-1024C24.1* and *MPPED2* in thyroid carcinoma cell lines. qRT-PCR performed on papillary (TPC-1, B-CPAP), follicular (WRO), anaplastic (FB-1 and FRO) thyroid carcinoma cell lines and three normal thyroid tissue samples (NT1, NT2, NT3). Data are reported as 2^−ΔCt^ values ± SD; Figure S3: Analysis of *RP5-1024C24.1* and *MPPED2* expression in TPC-1- and FRO-transfected cells. qRT-PCR analysis performed on TPC-1 and FRO cell lines stably expressing *RP5-1024C24.1* (**A**), *MPPED2* (**B**) or carrying the corresponding empty vector (EV), and three normal thyroid tissue samples (NT1, NT2, NT3). Data are reported as 2^−ΔCt^ values ± SEM; Table S1: PTC samples used for lncRNA microarray; Table S2: Whole list of lncRNAs deregulated in PTC vs. NT; Table S3: List of primer sequences used for qRT-PCR analyses.
