# Correction: Sepe et al. The Long Non-Coding RNA *RP5-1024C24.1* and Its Associated-Gene *MPPED2* Are Down-Regulated in Human Thyroid Neoplasias and Act as Tumour Suppressors. *Cancers* 2018, *10*, 146

**DOI:** 10.3390/cancers15154003

**Published:** 2023-08-07

**Authors:** Romina Sepe, Simona Pellecchia, Pierre Serra, Daniela D’Angelo, Antonella Federico, Maddalena Raia, Ricardo Cortez Cardoso Penha, Myriam Decaussin-Petrucci, Luigi Del Vecchio, Alfredo Fusco, Pierlorenzo Pallante

**Affiliations:** 1 Institute of Experimental Endocrinology and Oncology (IEOS) “G. Salvatore”, National Research Council (CNR), Via Sergio Pansini 5, 80131 Naples, Italy; romina.sepe@unina.it (R.S.);; 2 Department of Molecular Medicine and Medical Biotechnology (DMMBM), University of Naples “Federico II”, Via Sergio Pansini 5, 80131 Naples, Italy; 3 Service d’Anatomie et Cytologie Pathologiques, Centre de Biologie Sud, Groupement Hospitalier Lyon Sud, 69495 Pierre Bénite, France; 4 CEINGE-Biotecnologie Avanzate, Via Gaetano Salvatore 486, 80145 Naples, Italy; 5 Instituto Nacional de Cancer, Laboratorio de Carcinogênese Molecular, Rua Andre Cavalcanti 37, Centro, Rio de Janeiro 20231-050, Brazil

In the original publication [1], there was a mistake in Figure 4C as published. During the preparation of the original manuscript, we regret having made a careless error by reproducing the experiment of Figure 3C in Figure 4C (EV vs. RP5-1024C24.1 in the FRO cell line) instead of showing the EV vs. MPPED2 experiment in the FRO cell line. We have made a correction by providing a new panel for Figure 4C in the FRO cell line by assembling the right images for EV and MPPED2. The corrected Figure 4C appears below. The authors apologize for any inconvenience caused and state that the scientific conclusions are unaffected. This correction was approved by the Academic Editor. The original publication has also been updated.

## Figures and Tables

**Figure 4:**
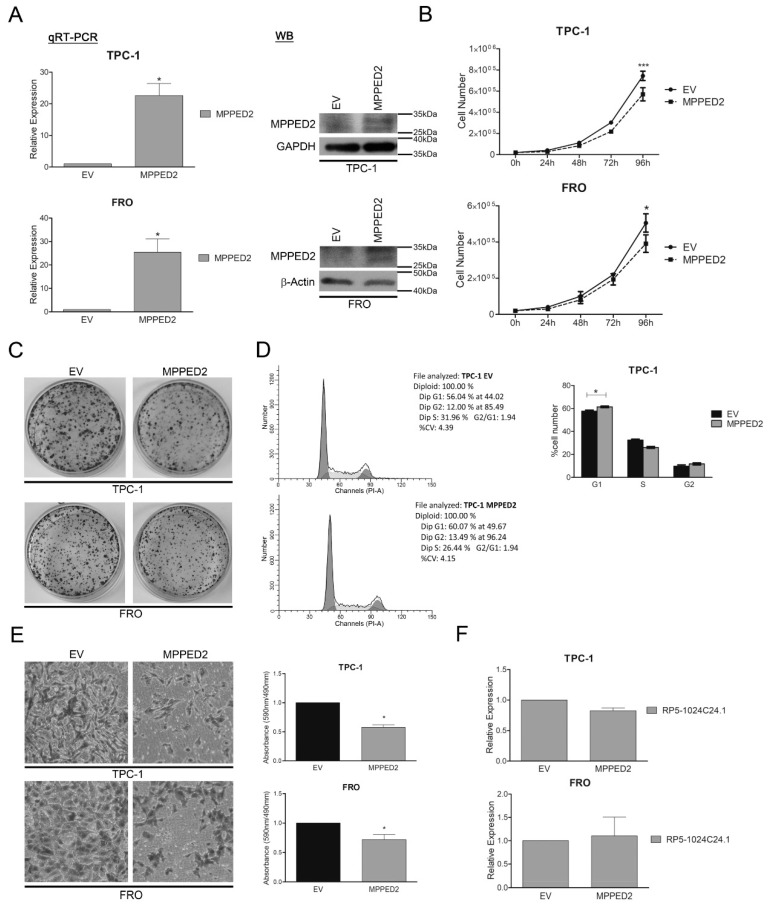
MPPED2 negatively modulates cell proliferation and migration of thyroid carcinoma cell lines. (**A**) qRT-PCR analysis performed on TPC-1 and FRO cell lines stably carrying *MPPED2* or the corresponding empty vector (*EV*). Values were reported as relative expression ± SEM and were compared to *EV*, set equal to 1. *t*-test; * *p* < 0.05 (left panel). Immunoblot analysis confirming the expression of MPPED2. GAPDH and β-Actin were used to normalize the amount of loaded protein (right panel). (**B**) Cell growth analysis of TPC-1 and FRO stably carrying *MPPED2* or *EV*. Cell number was evaluated at 24 h, 48 h, 72 h and 96 h after seeding. Values were obtained from three independent experiments performed in duplicate and data were reported as mean ± SEM. 2-way Anova-test followed by Bonferroni post-test; * p < 0.05; *** *p* < 0.001. (**C**) Representative colony assay performed on TPC-1 and FRO cell lines stably carrying *MPPED2* or *EV*. (**D**) Representative cell cycle analysis of TPC-1-*MPPED2* and TPC-1-*EV* cells. Cell number was reported on the *y*-axis while the percentage of propidium iodide (PI) incorporated was reported on the *x*-axis (left panel). Values shown in the right panel were obtained from three independent experiments. *t*-test; * *p* < 0.05 compared to *EV*. (**E**) Representative acquisition of migration assays performed on *MPPED2* or *EV* transfected TPC-1 and FRO cells (magnification 40×) (left panel). Values obtained from three (TPC-1) or four (FRO) independent experiments were reported as mean ± SEM and compared to the *EV*, set equal to 1 (right panel). *t*-test; * *p* < 0.05. (**F**) qRT-PCR analysis to evaluate the expression of *RP5-1024C24.1* after *MPPED2* transfection. Data obtained from three (TPC-1) or five (FRO) independent experiments were reported as relative expression ± SEM and were compared to the *EV*, set equal to 1. *t*-test; *p* = *ns*.
