# Supplementary material for: The Long Non-Coding RNA RP5-1024C24.1 and Its Associated-Gene MPPED2 Are Down-Regulated in Human Thyroid Neoplasias and Act as Tumour Suppressors
Source: Cancers (Basel). 2018 May 18;10(5):146. doi: 10.3390/cancers10050146 (PMC5977119; doi:10.3390/cancers10050146)
Supplement: Supplementary file 1 [file cancers-10-00146-s001.zip › Sepe et al_cancers-305832_Supplementary Material.doc]

**SUPPLEMENTARY MATERIAL**

**SUPPLEMENTARY FIGURES**

**Figure S1.** LncRNA expression analysis in additional PTC samples. qRT-PCR analysis to evaluate the expression of *RP11-230G5.2*, *DLEU2* and *SLC26A4-AS1* in additional PTC samples. Results are reported as relative expression ± SEM compared to the mean of three normal thyroid samples set equal to 1.

**Figure S2.** Expression analysis of *RP5-1024C24.1* and *MPPED2* in thyroid carcinoma cell lines. qRT-PCR performed on papillary (TPC-1, B-CPAP), follicular (WRO), anaplastic (FB-1 and FRO) thyroid carcinoma cell lines and three normal thyroid tissue samples (NT1, NT2, NT3). Data are reported as 2^-ΔCt^ values ± SD.

**A**

**B**

**Figure S3**. Analysis of *RP5-1024C24.1* and *MPPED2* expression in TPC-1- and FRO-transfected cells. qRT-PCR analysis performed on TPC-1 and FRO cell lines stably expressing *RP5-1024C24.1* (**A**), *MPPED2* (**B**) or carrying the corresponding empty vector (*EV)*, and three normal thyroid tissue samples (NT1, NT2, NT3). Data are reported as 2^-ΔCt^ values ± SEM.

**SUPPLEMENTARY TABLES**

**Table S1. PTC samples used for lncRNA microarray.**

| **Sample** | **Thyroid Tumour Hystotype^1^** | **Tumour Stage (TNM^2^)** |
| --- | --- | --- |
| *PTC1* | PTC-FV | pT1aN0 |
| *PTC2* | PTC-FV | pT2Nx |
| *PTC3* | PTC-FV | pT2aN0R0 |
| *PTC4* | PTC | pT2N0 |
| *PTC5* | PTC-FV | pT2Nx |
| *PTC6* | PTC-FV | pT2Nx |
| *PTC7* | PTC | pT3aNx |
| *PTC8* | PTC | pT2bN1 |
| *PTC9* | PTC | pT2bN0R0 |
| *PTC10* | PTC | pT2bN0R0 |
| *PTC11* | PTC-FV | pT2Nx |
| *PTC12* | PTC-FV | pT3Nx |

^1^ PTC, papillary thyroid carcinoma; FV, follicular variant.

^2^ TNM staging system: T, tumour size; N, regional lymph nodes metastasis; M, distant metastasis; R, relapse.

**Table S3.** List of primer sequences used for qRT-PCR analyses.

| **Primer name** | **Sequence 5'-3'** |
| --- | --- |
| *AC002066.1 Forward* | ACACATGAATGCCGAACCAC |
| *AC002066.1 Reverse* | CCAAGAAGACCCCGGTAAGAC |
| *AC008079.9 Forward* | GCACACCAACCAGTGTAGGA |
| *AC008079.9 Reverse* | AGAGTGGGCTGAAGCTTGTC |
| *AC079630.2 Forward* | TCCCTTGGCATATGGTGTGT |
| *AC079630.2 Reverse* | GCTTCCCAGTGTTTATCTCCA |
| *DLEU2 Forward* | GAAAGGTGTACTGCAAGGAACA |
| *DLEU2 Reverse* | GTACCGCACCTAGAGTAGGC |
| *EGFEM1P Forward* | GGCATGCCAAGTGTTTGTGA |
| *EGFEM1P Reverse* | AGTTCCCACTGCAGCGATAG |
| *G6PD Forward* | ACAGAGTGAGCCCTTCTTCAA |
| *G6PD Reverse* | ATAGGAGTTGCGGGCAAAG |
| *KHDRBS2 Forward* | TGTATTAATTGAAAGTGTTTGCTCCA |
| *KHDRBS2 Reverse* | CACGTAGTTGTTCCTGACGAAT |
| *LRRK2 Forward* | GAGCACGCCTCCAAGTTATT |
| *LRRK2 Reverse* | GCACAGAAGTGACCAACCCA |
| *MPPED2 Forward* | CGTTTCCCCTCTGTGTCCAA |
| *MPPED2 Reverse* | TTAAACCACGGGGTCCAAGG |
| *MSRB3 Forward* | GTGGAAACAAGCTGCTCTCA |
| *MSRB3 Reverse* | ACAAGGCAGCCGAATTTATG |
| *RP11-230G5.2 Forward* | AGAGCAGCCATTTGGAGGTA |
| *RP11-230G5.2 Reverse* | AGGAGGTTCTGGGTTGGTGA |
| *RP11-353N14.2 Forward* | TGTCTAACGCAGGGGGAAAC |
| *RP11-353N14.2 Reverse* | TATTGCCAACAACTGGCCCT |
| *RP1-240B8.3 Forward* | ACCTGTGACCTCACCTCAAA |
| *RP1-240B8.3 Reverse* | AGCCAGTCTTGGGTTTATTCCA |
| *RP5-1024C24.1 Forward* | TGGTGCAGGGATTGTTGCAT |
| *RP5-1024C24.1 Reverse* | TGAACGACTGCAACTGCTTTG |
| *SLC26A4 Forward* | ACAGAGCTGACGGAAGAAAA |
| *SLC26A4 Reverse* | CCATTTTGCCCATGTTAGGGAT |
| *SLC26A4-AS1 Forward* | ACCAGAACATTCTCGCTGA |
| *SLC26A4-AS1 Reverse* | TGCAAGGCAGGTGGATTACG |
| *ST7 Forward* | AAAGGGGCACCTATTTTATCCT |
| *ST7 Reverse* | TGGGTAAACTGAGACTTCATGG |
| *ST7-AS1 Forward* | CTCGCGTTCTGGGTCCATTT |
| *ST7-AS1 Reverse* | TGCCTGAGGCGTTTCCTTC |
| *TRIM13 Forward* | TCCATAATTCCAAAATCAGAAGC |
| *TRIM13 Reverse* | TTGAGCTTCAAACAATTGAATACC |
| *ZFY Forward* | ACACTGCACAGTCCCAGATG |
| *ZFY Reverse* | GGGCACAGTCTGCTACCAAT |
| *ZFY-AS1 Forward* | GAGTCCAGCAGAACAAAGTTGG |
| *ZFY-AS1 Reverse* | GCCTCACTTAGGGTTTCCAAG |
| *β-ACTIN Forward* | GAGCGCGGCTACAGCTT |
| *β-ACTIN Reverse* | TCCTTAATGTCACGCACGATTT |
